# Generalization of adding angular momenta and circular potential in quaternionic quantum mechanics

**DOI:** 10.1016/j.heliyon.2024.e25597

**Published:** 2024-02-05

**Authors:** R. Deepika, K. Muthunagai

**Affiliations:** School of Advanced Sciences, Vellore Institute Of Technology, Vandalur-Kelambakkam Road, Chennai, 600 127, Tamil Nadu, India

**Keywords:** Angular momentum, Anti-Hermitian operator, Circular potential, Quaternions

## Abstract

Complex numbers were created by introducing the imaginary unit *i* to represent the square root of -1, allowing solutions to equations that involved square roots of negative numbers. Complex numbers were further extended to quaternionic numbers by introducing additional imaginary units, namely “*j*” and “*k*”. Quaternions are non-commutative and involve four components, with each component being a real number or a multiple of an imaginary unit. Usually complex numbers are used to represent wave functions in quantum mechanics. Solutions using quaternions to square well, spin and angular momentum, Dirac equations have been obtained by many researchers. In this article, we have made use of quaternions to study the generalization of adding angular momenta, the digital signal processing of a quaternionic function and circular potential of a particle in real Hilbert space and have obtained quaternionic solutions in terms of Bessel functions.

## 1 Introduction

Quaternionic quantum mechanics is a generalized form of standard quantum mechanics that utilizes quaternions instead of complex numbers to characterize the wave functions and operators of quantum systems. Quaternions are four-dimensional entities that exhibit non-commutative multiplication and can inherently account for spin. This approach in quantum mechanics establishes connections with the Cauchy linear theory of elasticity and certain relativistic equations. In quantum mechanics, wave equations are used to describe the behavior of quantum systems in terms of wave functions that encode the probability of finding a particle in a given state. The most famous wave equation in quantum mechanics is the Schrödinger equation, which is a non-relativistic equation that relates the energy of a particle to its wave function. The Schrödinger equation can be written as:

$$i\hbar \frac{\partial \psi }{\partial t}=\overset{ˆ}{H}\psi$$

where *ψ* is the wave function, ‘*t*’ is the time, *ħ* is the reduced Planck's constant and $\overset{ˆ}{H}$ is the Hamiltonian operator that represents the total energy of the system. Wave functions that are used in quaternionic quantum mechanics state quantum mechanics is valued over quaternionic theory, i.e., Complex wave functions can be substituted by quaternionic functions.

The set of complex numbers has been extended to quaternionic numbers by introducing additional imaginary units *j* and *k* as follows.

q=r+iu+jv+kw

where r,u,v,w∈R, ${\text{i}}^{2}={\text{j}}^{2}={\text{k}}^{2}=-1$ and *ij*=*k*, *ji*=*-k* where *i*, *j*, *k* are anti-commutative imaginary unit vectors. This extension leads us from 2-dimensional complex system to a 4-dimensional quaternions and not to any 3-dimensional object. The anti-commutative property of imaginary units helps us to invert the quaternions to a non-commutative hypercomplex numbers. The collection of quaternionic numbers is represented by H. Hypothetically, if quanternionic numbers can substitute complex numbers then complex wave functions in quantum mechanics can be replaced using quaternionic wave functions.

In the initial approach, an anti-hermitian Hamiltonian operator has been applied to quaternionic quantum mechanics (HQM). Stephen Adler has discussed extensively a vast array of solutions utilizing this methodology [25]. On the other hand, anti-hermitian principles in quaternionic quantum mechanics have suffered from various drawbacks, notably the ill-defined classical limit. The interpretation of solutions within the framework of anti-hermitian HQM proves to be exceedingly challenging. Therefore, the disadvantages associated with anti-hermitian approaches hinder their efficacy in understanding and analyzing quantum systems.

Numerous studies have yielded results in this context [2], [3], [4], [5], [8], [9], [11], [14], [15], [16], [17], [18], [19], [20], [26], [27], [28], [29], [30], [31], [32], [33], [34]. For the applications of quaternionic numbers in quantum mechanics (HQM), one can refer [1], [6], [7], [10], [13], [22], [24]. Recently a different method to (HQM) eradicated the need of anti-hermiticity for the hamiltonian [35], [36] which has resulted in the proof of viral theorem [37] and has established a well defined classical limit [35]. It is evident that the stability of the real Hilbert space is at a higher level when compared to anti-hermitian HQM. Various results have been obtained using this method, including the Aharonov-Bohm effect [38], the behavior of free particles [39], [40], the quantum Lorentz law [37], and scattering phenomena [21]. These findings demonstrate the effectiveness of the real Hilbert space in addressing and explaining these physical phenomena.

There is ongoing research in the field of quaternionic quantum mechanics (HQM) that aims to study fundamental quantum systems. One interesting investigation involves the square-well potential, as discussed in a previous study [41]. The findings of this study suggest that certain modifications are necessary in terms of the configuration of the circular wave function, boundary conditions (BCs), and the extension of the quaternionic wave function into quaternionic Fourier series. Studying fundamental solutions in HQM is of great importance, as it provides a common foundation that can reconcile complex quantum mechanics (CQM) and HQM. These fundamental solutions can also serve as benchmarks for tackling more complex quaternionic systems in the future. By [42] spin and angular momentum in quaternionic quantum mechanics is proved.

In this article, we have obtained the solutions for generalization of adding angular momenta using quaternionic numbers and finding a solution for circular potential.

## 2 Practical applications of the angular momentum

The angular momentum in quaternionic quantum mechanics can be used in various applications in optics, imaging etc. The angular momentum of a particle is considered as the energy of the particle and the energy of the particle is transmitted as signals using complex valued functions.

For example, the representation of the quaternionic function

$f(t)=cos(t+2\pi )+e^{3t+2\pi }i+(2t+2\pi )j-(3t+2\pi )k$ in discrete signal processing with sampling frequency 165 Hz and the duration 25 sec is depicted in the following figure. With the understanding that the physical signal is the real part of *f* written as Re(*f*).

**Figure:**
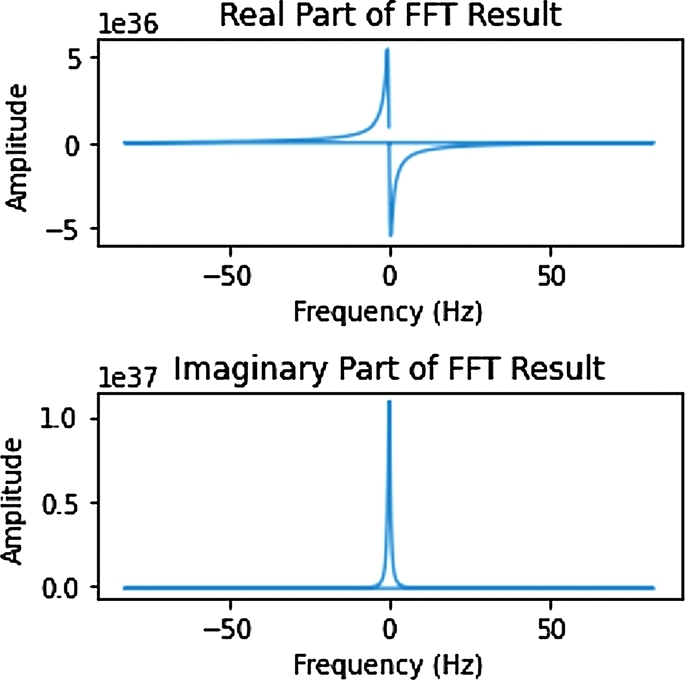

## 3 Addition of angular momentum in complex form

The given commutation relations for the triplet of Hermitian linear operators $\overset{ˆ}{G_{i}}$ on a quaternionic vector space *V* are:

$$[\overset{ˆ}{G_{i}},\overset{ˆ}{G_{j}}]=i\hbar \epsilon_{ijk}\overset{ˆ}{G_{k}}$$

Let us consider only two angular momenta:

Hermitian operators ${\overset{ˆ}{G}}_{i}^{\left(1\right)}$ on $V_{1}$ satisfying:

$$[{\overset{ˆ}{G_{i}}}^{\left(1\right)},{\overset{ˆ}{G_{j}}}^{\left(1\right)}]=i\hbar \epsilon_{ijk}{\overset{ˆ}{G_{k}}}^{\left(1\right)}$$

Hermitian operators ${\overset{ˆ}{G_{i}}}^{\left(2\right)}$ on $V_{1}$ satisfying:

$$[{\overset{ˆ}{G_{i}}}^{\left(2\right)},{\overset{ˆ}{G_{j}}}^{\left(2\right)}]=i\hbar \epsilon_{ijk}{\overset{ˆ}{G_{k}}}^{\left(2\right)}$$

To prove:

$${\overset{ˆ}{G}}_{i}\equiv {\overset{ˆ}{G_{k}}}^{\left(1\right)}⊗1+1⊗{\overset{ˆ}{G_{k}}}^{\left(2\right)}\text{ satisfies }[{\overset{ˆ}{G}}_{i},{\overset{ˆ}{G}}_{j}]=i\hbar \epsilon_{ijk}{\overset{ˆ}{G}}_{k}\text{ acting on }V_{1}⊗V_{2}$$

where $V_{1}⊗V_{2}$ is the tensor product of the quaternions in the vector spaces.

As the commutation holds, we have:

$$\left[\overset{ˆ}{G_{i}},\overset{ˆ}{G_{j}}]=[{\overset{ˆ}{G_{i}}}^{\left(1\right)}⊗1+1⊗{\overset{ˆ}{G_{i}}}^{\left(2\right)},{\overset{ˆ}{G_{j}}}^{\left(1\right)}⊗1+1⊗{\overset{ˆ}{G_{j}}}^{\left(2\right)}\right]$$

Neglecting the commutators of operators in different spaces, we have:

$$\left[\overset{ˆ}{G_{i}},\overset{ˆ}{G_{j}}]=[{\overset{ˆ}{G_{i}}}^{\left(1\right)},{\overset{ˆ}{G_{j}}}^{\left(1\right)}]⊗1+1⊗[{\overset{ˆ}{G_{i}}}^{\left(2\right)},{\overset{ˆ}{G_{j}}}^{\left(2\right)}\right]$$

Using independent algebras of angular momentum, we find:

$$[\overset{ˆ}{G_{i}},\overset{ˆ}{G_{j}}]=i\hbar \epsilon_{ijk}{\overset{ˆ}{G_{k}}}^{\left(1\right)}⊗1+i\hbar \epsilon_{ijk}1⊗{\overset{ˆ}{G_{k}}}^{\left(2\right)}$$

So,

$$[\overset{ˆ}{G_{i}},\overset{ˆ}{G_{j}}]=i\hbar \epsilon_{ijk}\overset{ˆ}{G_{k}}$$

Let *α* and *β* be two non-zero complex constants. Using these constants, we express:

$${\overset{ˆ}{G}}_{i}\equiv \alpha {\overset{ˆ}{G_{k}}}^{\left(1\right)}⊗1+\beta 1⊗{\overset{ˆ}{G_{k}}}^{\left(2\right)}$$

The commutator calculation yields:

$$\left[\overset{˜}{G_{i}},\overset{˜}{G_{j}}]=i\hbar \epsilon_{ijk}[\alpha^{2}{\overset{ˆ}{G_{k}}}^{\left(1\right)}⊗1+\beta^{2}1⊗{\overset{ˆ}{G_{k}}}^{\left(2\right)}\right]$$

The establishment of an angular momentum algebra hinges on the identification of the operator enclosed within the parentheses as $\overset{ˆ}{G_{k}}$. For this to hold, specific conditions must be $\alpha^{2}=\alpha$ and $\beta^{2}=\beta$. Given that neither *α* nor *β* can be zero, a singular solution arises if α=β=1. This compellingly verifies our adherence to the exclusive method of combining two angular momenta to yield a novel angular momentum entity.

This is an already existing result for two angular momenta in complex form.

## 4 Generalization of adding angular momenta in quaternionic form

A quaternionic non-commutative algebra is a four-dimensional associative normed division algebra over the set of real numbers, where the multiplication of the basis elements is not commutative. A quaternion can be written as a linear combination of the basis elements 1,*i*, *j*, *k* which satisfy the following relations: ${\text{i}}^{2}={\text{j}}^{2}={\text{k}}^{2}=\text{i}\text{j}\text{k}=-1$. An albeit non-commutative algebra is a more general term for an algebraic structure in which one of the principal binary operations is not commutative, that is, for which ab≠ba for some elements a and b.

Angular momentum is a concept in quantum mechanics that characterizes the rotational properties of particles. It's represented by a set of three anti-hermitian operators denoted as $\overset{ˆ}{G_{i}}$, which correspond to the x, y, and z components of angular momentum. These operators obey the commutation relations

$$[\overset{ˆ}{G_{i}},\overset{ˆ}{G_{j}}]=-i\hbar \epsilon_{ijk}\overset{ˆ}{G_{k}}$$

where $\epsilon_{ijk}$ is the Levi-Civita symbol and *ħ* is the reduced Planck's constant.

In this context, the angular momentum operators act on a quaternionic vector space denoted as *V*. A notable requirement is the absence of negatively normed states, which implies that the angular momentum operators define a unitary representation of the rotation group. This leads to the decomposition of the vector space *V* into sums of representations of angular momentum.

Let us assume for ‘n’ angular momenta:

Anti-Hermitian operators ${\overset{ˆ}{G_{i}}}^{\left(1\right)}$ on $V_{1}$ satisfying:

$$[{\overset{ˆ}{G_{i}}}^{\left(1\right)},{\overset{ˆ}{G_{j}}}^{\left(1\right)}]=-i\hbar \epsilon_{ijk}{\overset{ˆ}{G_{k}}}^{\left(1\right)}$$

Anti-Hermitian operators ${\overset{ˆ}{G_{i}}}^{\left(2\right)}$ on $V_{2}$ satisfying:

$$[{\overset{ˆ}{G_{i}}}^{\left(2\right)},{\overset{ˆ}{G_{j}}}^{\left(2\right)}]=-i\hbar \epsilon_{ijk}{\overset{ˆ}{G_{k}}}^{\left(2\right)}$$

…

Anti-Hermitian operators ${\overset{ˆ}{G_{i}}}^{\left(n\right)}$ on $V_{n}$ satisfying:

$$[{\overset{ˆ}{G_{i}}}^{\left(n\right)},{\overset{ˆ}{G_{j}}}^{\left(n\right)}]=-i\hbar \epsilon_{ijk}{\overset{ˆ}{G_{k}}}^{\left(n\right)}$$

Our assertion is that the angular momenta sum to form an angular momentum within the tensor product.

Case 1 For n=Odd

$$\overset{ˆ}{G_{i}}=\left[{\overset{ˆ}{G_{i}}}^{\left(1\right)}⊗1+{\overset{ˆ}{G_{i}}}^{\left(3\right)}⊗1+\ldots +{\overset{ˆ}{G_{i}}}^{\left(n\right)}⊗1,{\overset{ˆ}{G_{i}}}^{\left(1\right)}⊗1+{\overset{ˆ}{G_{i}}}^{\left(3\right)}⊗1+\ldots +{\overset{ˆ}{G_{i}}}^{\left(n\right)}⊗1\right]$$

This leads to:

$$\overset{ˆ}{G_{i}}=-i\hbar \epsilon_{ijk}\overset{ˆ}{G_{k}}\;\text{acting on }V_{1}⊗V_{3}⊗\ldots ⊗V_{n}$$

The summation operator, as previously defined, operates on the tensor product space $V_{1}⊗V_{2}⊗\ldots ⊗V_{n}$. Notably, it possesses the property of being an anti-Hermitian operator within this tensor product space.

Our primary objective is to verify the validity of the commutation relation:

$$\left[\overset{ˆ}{G_{i}},\overset{ˆ}{G_{j}}]=[{\overset{ˆ}{G_{i}}}^{\left(1\right)}⊗1+{\overset{ˆ}{G_{i}}}^{\left(3\right)}⊗1+\ldots +{\overset{ˆ}{G_{i}}}^{\left(n\right)}⊗1,{\overset{ˆ}{G_{j}}}^{\left(1\right)}⊗1+{\overset{ˆ}{G_{j}}}^{\left(3\right)}⊗1+\ldots +{\overset{ˆ}{G_{j}}}^{\left(n\right)}⊗1\right]$$

The mixed terms representing commutators among operators in distinct spaces vanish. Therefore we deduce: $\left[\overset{ˆ}{G_{i}},\overset{ˆ}{G_{j}}]=[{\overset{ˆ}{G_{i}}}^{\left(1\right)}⊗1,{\overset{ˆ}{G_{j}}}^{\left(1\right)}⊗1]+[{\overset{ˆ}{G_{i}}}^{\left(1\right)}⊗1,{\overset{ˆ}{G_{j}}}^{\left(3\right)}⊗1]+[{\overset{ˆ}{G_{i}}}^{\left(3\right)}⊗1,{\overset{ˆ}{G_{j}}}^{\left(5\right)}⊗1]+\ldots +[{\overset{ˆ}{G_{i}}}^{\left(n\right)}⊗1,{\overset{ˆ}{G_{j}}}^{\left(n\right)}⊗1\right]$. Now, utilizing the concept of independent angular momentum algebras, we can determine:

$$[\overset{ˆ}{G_{i}},\overset{ˆ}{G_{j}}]=-i\hbar \epsilon_{ijk}{\overset{ˆ}{G_{k}}}^{\left(1\right)}⊗1-i\hbar \epsilon_{ijk}{\overset{ˆ}{G_{k}}}^{\left(3\right)}⊗1-\ldots -i\hbar \epsilon_{ijk}{\overset{ˆ}{G_{k}}}^{\left(n\right)}⊗1$$

Hence,

(1) $$\left[\overset{ˆ}{G_{i}},\overset{ˆ}{G_{j}}]=-i\hbar \epsilon_{ijk}[{\overset{ˆ}{G_{k}}}^{\left(1\right)}⊗1+{\overset{ˆ}{G_{k}}}^{\left(3\right)}⊗1+\ldots +\overset{ˆ}{G_{k}^{\left(n\right)}}⊗1\right]$$

Case 2 For *n* = Even

$$\overset{ˆ}{G_{i}}=\left[1⊗{\overset{ˆ}{G_{i}}}^{\left(2\right)}+1⊗{\overset{ˆ}{G_{i}}}^{\left(4\right)}+\ldots +1⊗{\overset{ˆ}{G_{i}}}^{\left(n\right)},1⊗{\overset{ˆ}{G_{i}}}^{\left(2\right)}+1⊗{\overset{ˆ}{G_{i}}}^{\left(4\right)}+\ldots +1⊗{\overset{ˆ}{G_{i}}}^{\left(n\right)}\right]$$

$$\overset{ˆ}{G_{i}}=-i\hbar \epsilon_{ijk}\overset{ˆ}{G_{k}}\text{ acting on }V_{2}⊗V_{4}⊗\ldots ⊗V_{n}.$$

The sum operator, as previously defined, functions as an operator on $V_{2}⊗V_{4}⊗...⊗V_{n}$. It is, in fact, an anti-Hermitian operator on $V_{2}⊗V_{4}⊗...⊗V_{n}$.

Our objective is to verify the validity of the commutation relation:

$$[\overset{ˆ}{G_{i}},\overset{ˆ}{G_{j}}]=\left[1⊗{\overset{ˆ}{G_{i}}}^{\left(2\right)},1⊗{\overset{ˆ}{G_{j}}}^{\left(2\right)}\right]+\left[1⊗{\overset{ˆ}{G_{i}}}^{\left(4\right)},1⊗{\overset{ˆ}{G_{j}}}^{\left(4\right)}\right]+\ldots +\left[1⊗{\overset{ˆ}{G_{i}}}^{\left(n\right)},1⊗{\overset{ˆ}{G_{j}}}^{\left(n\right)}\right]$$

Considering that the mixed terms, indicative of commutators involving operators from distinct spaces, become zero, we conclude:

$$\left[\overset{ˆ}{G_{i}},\overset{ˆ}{G_{j}}]=1⊗[{\overset{ˆ}{G_{i}}}^{\left(2\right)},{\overset{ˆ}{G_{j}}}^{\left(2\right)}]+1⊗[{\overset{ˆ}{G_{i}}}^{\left(4\right)},{\overset{ˆ}{G_{j}}}^{\left(4\right)}]+\ldots +1⊗[{\overset{ˆ}{G_{i}}}^{\left(n\right)},{\overset{ˆ}{G_{j}}}^{\left(n\right)}\right]$$

Using the independent algebra of angular momentum, we find:

$$[\overset{ˆ}{G_{i}},\overset{ˆ}{G_{j}}]=-i\hbar \epsilon_{ijk}1⊗{\overset{ˆ}{G_{k}}}^{\left(1\right)}-i\hbar \epsilon_{ijk}1⊗{\overset{ˆ}{G_{k}}}^{\left(2\right)}-\ldots -i\hbar \epsilon_{ijk}1⊗{\overset{ˆ}{G_{k}}}^{\left(n\right)}$$

(2) $$\left[\overset{ˆ}{G_{i}},\overset{ˆ}{G_{j}}]=-i\hbar \epsilon_{ijk}[1⊗{\overset{ˆ}{G_{k}}}^{\left(2\right)}+1⊗{\overset{ˆ}{G_{k}}}^{\left(4\right)}+\ldots +1⊗{\overset{ˆ}{G_{k}}}^{\left(n\right)}\right]$$

Adding equations (1) and (2), we get:

$$\left[\overset{ˆ}{G_{i}},\overset{ˆ}{G_{j}}]=-i\hbar \epsilon_{ijk}[{\overset{ˆ}{G_{k}}}^{\left(1\right)}⊗1+{\overset{ˆ}{G_{k}}}^{\left(3\right)}⊗1+...+{\overset{ˆ}{G_{k}}}^{\left(n\right)}⊗1+1⊗{\overset{ˆ}{G_{k}}}^{\left(2\right)}+1⊗{\overset{ˆ}{G_{k}}}^{\left(4\right)}+...+1⊗{\overset{ˆ}{G_{k}}}^{\left(n\right)}\right]$$

$$[\overset{ˆ}{G_{i}},\overset{ˆ}{G_{j}}]=-i\hbar \epsilon_{ijk}\overset{ˆ}{G_{k}}$$

Let us use *n* non-zero real constants $\alpha_{1}$, $\alpha_{2},...,\alpha_{n}$ for the odd case, and $\beta_{1}$, $\beta_{2}...,\beta_{n}$ for the even case.

The commutator calculation yields:

$\left[\overset{ˆ}{G_{i}},\overset{ˆ}{G_{j}}]=-i\hbar \epsilon_{ijk}[\alpha_{1}^{2}{\overset{ˆ}{G_{k}}}^{\left(1\right)}⊗1+\alpha_{2}^{2}{\overset{ˆ}{G_{k}}}^{\left(3\right)}⊗1+...+\alpha_{n}^{2}{\overset{ˆ}{G_{k}}}^{\left(n\right)}⊗1+\beta_{1}^{2}1⊗{\overset{ˆ}{G_{k}}}^{\left(2\right)}+\beta_{2}^{2}1⊗{\overset{ˆ}{G_{k}}}^{\left(4\right)}+...+\beta_{n}^{2}1⊗{\overset{ˆ}{G_{k}}}^{\left(n\right)}\right]$

$$[\overset{ˆ}{G_{i}},\overset{ˆ}{G_{j}}]=-i\hbar \epsilon_{ijk}\overset{ˆ}{G_{k}}$$

The establishment of an angular momentum algebra hinges on the identification of the operator enclosed within the parentheses as $\overset{ˆ}{G_{k}}$ for this to hold, specific conditions must be “$\alpha^{2}=\alpha$ and $\beta^{2}$ = *β*”. Given that neither *α* nor *β* can be zero a singular solution arises if α=β=1. This compellingly verifies our adherence to the exclusive method of combining two angular momenta to yield a novel angular momentum entity. This confirms that we are using the unique way to add ‘n’ angular momenta to form a new “angular momentum”.

## 5 Particle on a ring

Following is the quaternionic Schrödinger equation for the wavefunction Ψ(l) with the time independent potential energy *V*.

(3) $$\hbar \frac{\partial \Psi }{\partial t}i=(-\frac{\hbar^{2}}{2m}\frac{\partial^{2}\Psi }{\partial l^{2}}+V(l))\Psi (l)$$

where *i* is the imaginary unit Ψ(l) is the quaternionic wave function with respect to ‘*l*’ and the Hamiltonian operator is with time independent potential V(l). The time derivative of the wave function is multiplied by the imaginary unit, while the Hamiltonian remains unchanged in case of a complex wave function. Despite its clarity, a study on circular ring potential has been carried out in quantum mechanics.

Consider a particle confined to a circle of circumference R. The coordinate along the circle is called *l* and we can view the circle as the interval *l* ∈[0,2π]. We know that Ψ is periodic and so

(4) Ψ(l+2π)=Ψ(l).

It is to be noticed that not only Ψ and all of its derivatives are also periodic.

### 5.1 Quaternionic solution

The time-independent results for (4) are given by

$$\Psi (l)=N\left(\cos(kl)e^{i\phi_{0}}+j\sin(kl)e^{i\xi_{0}}\right),\;N\in R,\;l,\phi_{0},\xi_{0}\in [0,2\pi ].$$

The normalization constant is real, which aligns with the real Hilbert space, as discussed in [35][36]. The proposal of such a wave function as the basis element of the quaternionic Fourier series was initially introduced in [36]. Remarkably, there exists an intriguing similarity between the complex and quaternionic cases. Using the periodicity condition we get

kl=2nπ

Therefore the normalization constant is calculated by $\int_{0}^{2\pi }$$[\Psi^{⁎}\Psi ]dl$ = 1

$$N=\frac{1}{\sqrt{2\pi }}$$

The steady state elements are made up of basis elements that posses the orthogonality property:

$$\int_{0}^{2\pi }\Psi_{k}{\Psi^{⁎}}_{k^{\prime }}dl=\delta_{kk^{\prime }}$$

where we used

$$\Psi_{k}\Psi_{k^{\prime }}^{⁎}=\frac{1}{2\pi }\left(\cos(k-k^{\prime })le^{i(\phi_{0}+\xi_{0})}+j\sin(k-k^{\prime })le^{i(\phi_{0}+\xi_{0})}\right)$$

*l* ∈ [0,2*π*.]

In [36], we find the expectation value of the quaternionic wave function in real Hilbert space given by

$$<T>=\frac{1}{2}\int [T\Psi ]\Psi^{⁎}+\Psi [T\Psi^{⁎}]dl^{3}$$

where T is the any arbitrary quaternionic operator on Hilbert spaces and the expectation value that we get here is not the usual case. This is a small novel solution that is calculated using quaternionic numbers. This type of solution differs with the usual solution in the dimensions. This type of solution gives the expectation value of the particles in a particular interval with two or four dimensions.

## 6 Infinite 2-D circular potential

### 6.1 Quaternionic solution

The quaternionic solutions for the infinite circle using the unitary basis of real Hilbert space is

$$\Psi_{nl}(\rho ,\theta )=C_{nl}J_{\left|l\right|}(k\rho )\left[\cos(l\theta )e^{i\phi_{0}}+j\sin(l\theta )e^{i\xi_{0}}\right]$$

where $C_{nl}$ is a constant, $J_{\left|l\right|}(k\rho )$ is the bessel function and $\theta ,\phi_{0},\xi_{0}\in [0,2\pi ]$. The steady state elements comprise of basis elements that posses the orthogonality property.

$$\int_{0}^{2\pi }\Psi_{nl}\Psi_{n^{\prime }l^{\prime }}^{⁎}d\theta =\delta_{nn^{\prime }}\delta_{ll^{\prime }}$$

in which we have used

$$\Psi_{nl}\Psi_{n^{\prime }l^{\prime }}^{⁎}=C_{n^{\prime }l^{\prime }}C_{nl}J_{\left|l\right|}(k\rho )J_{\left|l^{\prime }\right|}(k^{\prime }\rho^{\prime })\left[\cos(l-l^{\prime })\theta +j\sin(l-l^{\prime })\theta e^{i(\phi_{0}+\xi_{0})}\right]$$

The expectation value of quaternionic wave function in a real Hilbert space is defined as:

$$<T>=\frac{1}{2}\int [T\Psi ]\Psi^{⁎}+\Psi [T\Psi^{⁎}]dl^{3}$$

T is any arbitrary Hilbert space operator. The quaternionic circular potential well is non-restrictive. This is the advantage of circular potential well over square potential well.

Remark *If* $\phi_{0}=\xi_{0}$ *, on applying the periodicity condition of circular potential will lead us to the existing complex solutions as stated in* *[12]* *.*

## 7 Conclusion

In this paper, we present the generalization of adding angular momenta and quaternionic form for a circular potential well in finite and infinite 4 dimensions. This gives the solution in real Hilbert spaces and its Fourier series in quaternionic form. Quaternionic solution has topological differences when compared with complex solution. One of the major applications of this work is that it can be used to find the position of the particle in 4-Dimensions with a given radius and angle and which can also be useful in making complicated models. One of the possibilities are the shadows and photon spheres of black holes surrounded by spherical accretions in the four-dimensional Einstein-Gauss-Bonnet gravity [23], [43]. Quaternionic solutions reveals interesting properties of the polynomial equations, such as the number and nature of the roots.

## CRediT authorship contribution statement

**R. Deepika:** Writing – original draft. **K. Muthunagai:** Writing – review & editing.

## Declaration of Competing Interest

The authors declare the following financial interests/personal relationships which may be considered as potential competing interests: K. Muthunagai reports was provided by 10.13039/501100004728Vellore Institute of Technology - Chennai Campus. K. Muthunagai reports a relationship with Vellore Institute of Technology - Chennai Campus that includes: employment. If there are other authors, they declare that they have no known competing financial interests or personal relationships that could have appeared to influence the work reported in this paper.

## Data Availability

No data was used for the research described in the article.
